# The association between clinical and biochemical characteristics of late-onset sepsis and bronchopulmonary dysplasia in preterm infants

**DOI:** 10.1007/s00431-021-03981-9

**Published:** 2021-02-25

**Authors:** Melania E. Ebrahimi, Michelle Romijn, Roos J. S. Vliegenthart, Douwe H. Visser, Anton H. van Kaam, Wes Onland

**Affiliations:** 1 Department of Neonatology, Emma Children’s Hospital, Amsterdam UMC, University of Amsterdam, Vrije Universiteit Amsterdam, Amsterdam, The Netherlands; 2 Amsterdam Reproduction & Development Research Institute, Amsterdam UMC, University of Amsterdam, Vrije Universiteit Amsterdam, Amsterdam, The Netherlands

**Keywords:** Premature infants, Late-onset sepsis, Bronchopulmonary dysplasia

## Abstract

Studies in preterm infants have shown an association between late-onset sepsis (LOS) and the development of bronchopulmonary dysplasia (BPD). It is unknown whether clinical or biochemical characteristics during sepsis modulate the risk for BPD. This single-center retrospective cohort study included all patients with a gestational age < 30 weeks, born between 2009 and 2015, in whom empiric antimicrobial treatment was initiated > 72 h after birth and continued for at least 5 days, independent on microbiological results. The association between clinical and biochemical characteristics of LOS and the development of BPD in survivors were assessed with multivariate logistic regression analysis adjusted for early-onset sepsis, small for gestational age, and gestational age. Of the 756 admitted infants, 256 infants (mean GA: 27.0 weeks; birthweight: 924 grams) had at least one LOS episode, of whom 79 (30.9%) developed BPD. Analyses showed that only the need for and duration of mechanical ventilation during LOS were independently associated with an increased risk for BPD (adjusted OR 2.62, 95% CI 1.38, 4.96, *p* value 0.003, and OR 1.004, 95% CI 1.00, 1.007, *p* value 0.045, respectively).

*Conclusion:* During a LOS, the need for and duration of mechanical ventilation are independently associated with the risk of developing BPD in preterm infants.

**Table:** 

**What is Known:** • *Premature infants diagnosed with a late-onset sepsis are at higher risk of developing bronchopulmonary dysplasia* • *This association is mainly shown in infants with a positive blood culture*	
**What is New:** • *This study investigates the clinical and biochemical characteristics of late-onset sepsis and the development of bronchopulmonary dysplasia* • *The need for mechanical ventilation and duration of mechanical ventilation during late-onset sepsis are associated with an increased risk of developing bronchopulmonary dysplasia.*

**Supplementary Information:**

The online version contains supplementary material available at 10.1007/s00431-021-03981-9.

## Introduction

Bronchopulmonary dysplasia (BPD) remains the most common complication in extremely preterm born infants [1, 2]. While the pathogenesis is multifactorial and complex, inflammation plays a central role in the development of BPD [3, 4]. Systemic inflammation due to neonatal sepsis interacts with lung development causing underdeveloped alveoli and dysmorphic pulmonary vasculature, which are the histological hallmarks of BPD [5–8]. The association between sepsis and the risk for developing BPD is well established [5, 9, 10]. Observational studies have shown that both early-onset neonatal sepsis (EOS; < 72 h after birth) and late-onset neonatal sepsis (LOS; ≥ 72 h after birth) are associated with an increased risk for mortality and BPD [9–13]. However, most of these studies only included infants with culture-proven sepsis, while a negative blood culture does not exclude sepsis in this population [14–16]. Furthermore, these studies did not consider the fact that sepsis is a complex systemic disease with distinct differences in causative microorganism, age at onset, and clinical or biochemical characteristics. To date it remains unclear if and how these differences impact the association between sepsis and BPD. Unraveling which sepsis characteristics are independently associated with the development of BPD might improve our ability to identify those patient most at risk for developing BPD and target treatment to this specific population.

To address the abovementioned knowledge gaps, the current study collected data on the characteristics of sepsis and the diagnosis BPD in a population of preterm infants treated with antibiotics for clinical or culture-proven sepsis. Given the difference in incidence and clinical/biochemical characteristics between early and late-onset sepsis, we focused in this study on episodes of LOS [17]. The aim of this study is to investigate if the development of BPD is associated with one or more clinical and/or biochemical characteristics of sepsis.

## Materials and methods

### Study design, setting, and participants

This retrospective observational cohort study included preterm infants below 30 weeks of gestation who were admitted within 24 h after birth to the Neonatal Intensive Care Unit (NICU) of the Amsterdam University Medical Centers, between January 1, 2009, and December 31, 2015. Infants were eligible if empiric antimicrobial treatment was initiated ≥ 72 h after birth and continued for at least 5 days independent of microbiological results. For parental consent, an opt-out procedure was followed, and this study was approved by the institutional review board.

### Data collection

The following characteristics and outcomes were collected for each patient: sex; gestational age (GA); birthweight (BW); small for gestational age (SGA) defined as BW below the 10th percentile [18]; outborn; singleton birth; administration of antenatal steroids; mode of delivery; premature rupture of the membranes; Apgar score at 5 min; mode and duration of respiratory support in the delivery room and NICU; ventilation free days during admission; base FiO2 and respiratory support before LOS episode; treatment with exogenous surfactant; and caffeine and dexamethasone.

Early-onset sepsis (EOS) was defined as an infectious episode starting within 72 h after birth for which antibiotic treatment was given for 5 days or more. Late-onset sepsis (LOS) was defined as an episode with clinical signs and symptoms suspected for sepsis starting ≥ 72 h after birth for which empiric antimicrobial treatment was initiated and continued for at least 5 days independent of microbiological results. In this group, we also included infants with NEC ≥ grade 2, since the majority of these patients has a culture-proven or a clinical-suspected sepsis episode [19].

For every episode of LOS, the following data were collected: postnatal age of first administration of antibiotics, divided in 3–7 days, 7–14 days, and > 14 days after birth; the number of LOS episodes; type of microorganism; c-reactive protein (CRP); white blood cell count; use of cardiotonics (dopamin, dobutamin, epinephrine, or norepinephrine); and need for and duration of mechanical ventilation during LOS episode.

The primary outcome of this study was moderate or severe BPD defined as the need for supplemental oxygen for at least 28 days and the need for ≥ 30% supplemental oxygen or positive pressure ventilation at 36-week postmenstrual age (PMA) [20].

### Data analysis

Statistical analysis was performed with IBM SPSS Statistics for Windows, version 24 (IBM Corp., Armonk, NY, USA). Data were expressed as mean with standard deviation (SD) or median with interquartile range (IQR) depending on their distribution. Patients with missing BPD diagnoses and those infants who died before 36 weeks PMA were excluded from the analyses. Whole case analysis was performed. Patient characteristics were compared between infants with and without BPD with univariate analyses, e.g., Chi square-tests for dichotomous data and independent samples *t* tests for continuous data or their nonparametric equivalents.

To explore the association between LOS-associated factor, such as the postnatal age of first administration of antibiotics, number of LOS episodes, laboratory findings, medication use and mechanical ventilation, and the incidence of BPD, logistic regression analyses were performed. In case of multiple LOS episodes, the LOS-associated factors of the most severe infectious episode were used, defined as the episode with most of the following conditions present: an elevated CRP > 45 mg/L [21], leukopenia (defined as < 4 × 10^9/L) or leukocytosis (defined as > 20 × 10^9/L) [22], use of cardiotonics, and need for mechanical ventilation. A multivariate regression model was performed to investigate whether this association was influenced by EOS, GA, and SGA since these factors considered as independent risk factors for a LOS episode [9, 23]. To assess model fit, the Hosmer-Lemeshow goodness-of-fit test was performed.

Two sensitivity analyses were performed to test the robustness of possible associations between LOS characteristics and BPD. First, we assessed the impact of having a positive blood culture, by excluding infants with culture negative sepsis. Second, we assessed the impact of having multiple episodes of LOS by including only infants enduring one LOS episode. Data were expressed as odds ratios (OR) and 95% confidence interval (CI). A *p* value of < 0.05 was considered statistical significant.

## Results

### Patient characteristics

**Fig. 1:**
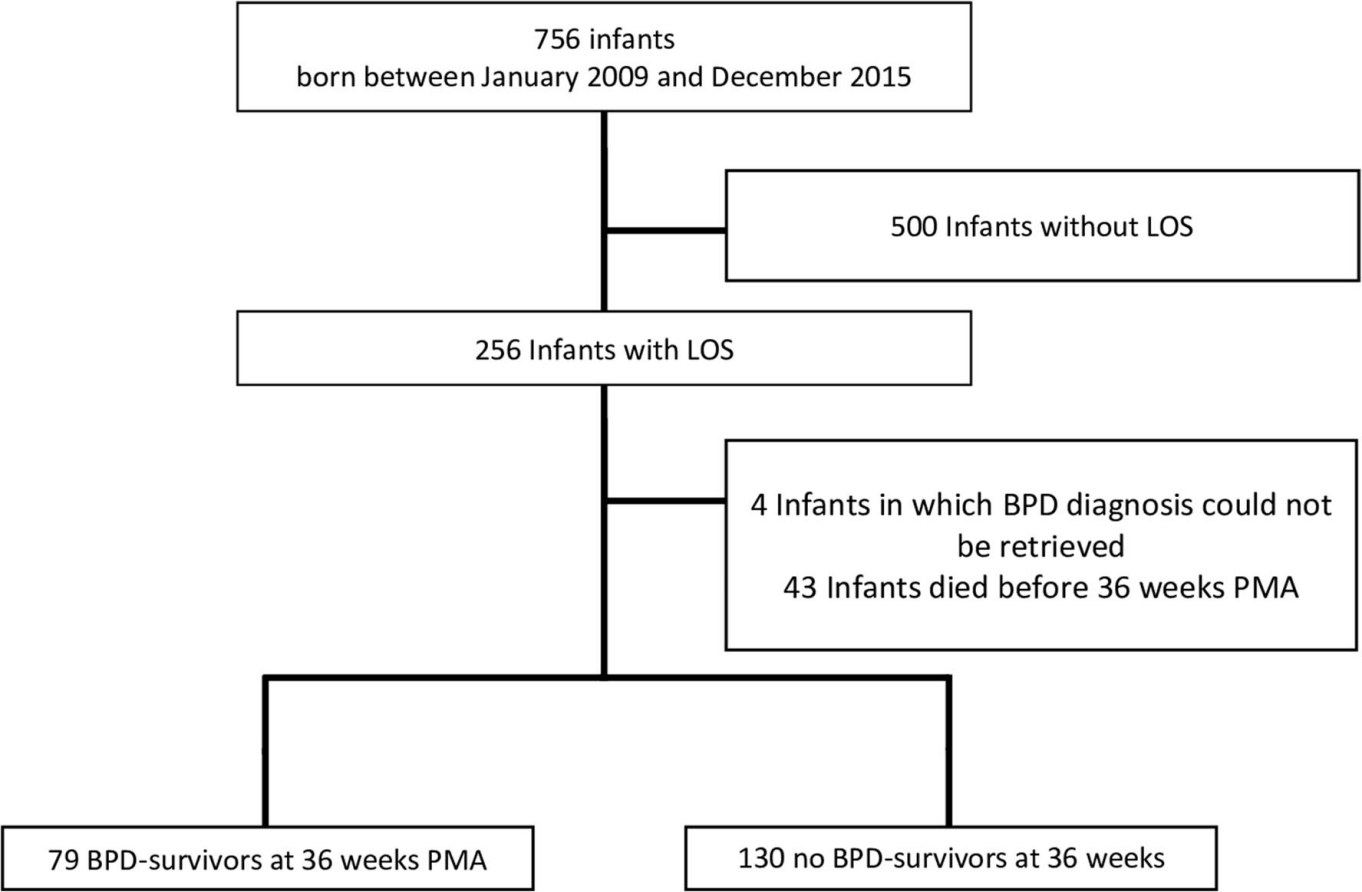
Flow chart. LOS: late onset sepsis. PMA: postmenstrual age. BPD: Bronchopulmonary dysplasia. NEC: Necrotizing enterocolitis.

Of the 756 infants admitted during the study period, 256 (33.8%) infants were diagnosed with one or more LOS episode during hospitalization. Compared to the infants without LOS, the infants with LOS had lower BW, GA, and a higher incidence of all major morbidities such as PDA, NEC, and BPD (Supplemental Table 1). BPD diagnosis could not be retrieved in 4 cases (1.5%). Forty three (16.7%) of the infants with one or more LOS episodes died before 36 weeks PMA (Fig. 1). Therefore, the final cohort of interest consisted of 209 infants, of whom 79 (37.8%) developed moderate to severe BPD at 36 weeks PMA. The patient characteristics of the survivors with or without BPD are summarized in Table 1. Compared to the infants without BPD, the infants diagnosed with BPD had a lower GA, BW, longer duration of (non-)invasive respiratory support, and were more often treated with surfactant, and dexamethasone.

**Table 1:** Patient characteristics of preterm survivors with and without BPD at 36-week postmenstrual age

Characteristics	BPD *n* = 79	No BPD *n* = 130	*p* value
Gender, male—no. (%)	49 (62.0%)	65 (50.0%)	0.90
Gestational age—wk median/IQR	26.7 (25.7, 27.9)	27.6 (26.1, 28.9)	< 0.001
Birth weight—g mean/SD	861 (200)	983 (263)	< 0.001
Small for gestational age—no. (%)	19 (24.1%)	18 (13.8%)	0.06
Outborn—no. (%)	6 (7.6%)	10 (7.7%)	0.98
Singleton—no. (%)	58 (73.4%)	94 (72.3%)	0.86
Antenatal corticosteroids—no. (%)	74 (93.7%)	120 (92.3%)	0.71
Cesarean section—no. (%)	34 (43.0%)	61 (46.9%)	0.58
PPROM—no. (%)	18 (22.8%)	25 (19.2%)	0.54
Apgar score at 5 min median/IQR	8 (6, 9)	8 (7, 9)	0.51
EOS—no. (%)	27 (34.2%)	31 (23.8%)	0.11
Intubation at delivery room—no. (%)	12 (15.2%)	11 (8.5%)	0.43
Mechanical ventilation during admission—no. (%)	71 (89.9%)	95 (73.1%)	< 0.001
Mechanical ventilation (days) median/IQR	8.3 (2.9, 16.3)	2.6 (1.4, 6.1)	< 0.001
CPAP (days) median/IQR	40.5 (32.4, 46.0)	27.8 (13.1, 39.0)	< 0.001
Ventilation free during admission (days) mean/SD	58.6 (23.9)	39.3 (17.0)	< 0.001
Base FiO2 before mechanical ventilation during LOS—median/IQR	0.37 (0.26, 0.55)	0.25 (0.21, 0.51)	0.30
Respiratory support before mechanical ventilation during LOS			
High flow—no. (%)	0 (0.0%)	6 (9.1%)	
CPAP—no. (%)	43 (74.1%)	42 (63.6%)	0.09
nIMV—no. (%)	15 (25.9%)	12 (18.2%)	
Surfactant—no. (%)	48 (60.8%)	55 (42.3%)	0.01
Caffeine—no. (%)	78 (98.7%)	128 (98.5%)	0.87
Dexamethasone—no. (%)	19 (24.1%)	10 (7.7%)	< 0.001

*PPROM* preterm premature rupture of membranes, *EOS* early-onset sepsis, *CPAP* continuous positive airway pressure. *IQR* interquartile range, *no* number, *SD* standard deviation

### Outcome

The need for and duration of mechanical ventilation during LOS are higher in the group of infants developing BPD, compared to the group of infants without BPD (OR 3.22, 95% CI 1.76, 5.91, *p* value < 0.001, and OR 1.004, 95% CI 1.001, 1.007, *p* value 0.023) (Table 2). The postnatal age of LOS onset, the number of LOS episodes, the type of microorganism found in the blood culture, a CRP value > 45 mg/L [21], leukocytosis or leukopenia, and use of cardiotonics were not different in the BPD versus no BPD group. The multivariate logistic regression analysis correcting for EOS, GA, and SGA showed that only the need for and duration of mechanical ventilation during LOS were independently associated with an increased risk for BPD (adjusted OR 2.62, 95% CI 1.38, 4.96, *p* value 0.003, and OR 1.004, 95% CI 1.00, 1.007, *p* value 0.045)**.**

**Table 2:** Late-onset sepsis characteristics in preterm survivors with and without BPD at 36-week postmenstrual age

Infants with LOS	BPD *N* = 79	No BPD *N* = 130	OR	95% CI	*p* value	aOR^a^	95% CI^a^	*p* value^a^	Hosmer-Lemeshow
Postnatal age 1st LOS onset									
< 7 days—no. (%)	22 (27.8%)	43 (33.1%)	0.78	(0.42, 1.44)	0.43	0.83	(0.42, 1.62)	0.58	ns
7–14 days—no. (%)	33 (41.8%)	59 (45.4%)	0.86	(0.49, 1.52)	0.61	0.87	(0.48, 1.58)	0.65	ns
≥ 14 days—no.(%)	24 (30.4%)	28 (21.5%)	1.59	(0.84, 3.00)	0.15	1.45	(0.74, 2.84)	0.28	ns
> 1 episode of LOS—no. (%)	28 (35.4%)	32 (24.6%)	1.68	(0.91, 3.09)	0.095	1.27	(0.65, 2.48)	0.49	0.030
Positive blood culture—no.(%)	59 (74.7%)	100 (76.9%)	0.89	(0.46, 1.70)	0.71	0.95	(0.48, 1.87)	0.88	ns
Gram stained bacteria									
Gram pos—no. (%)	24 (30.4%)	40 (30.8%)	0.98	(0.54, 1.80)	0.95	0.82	(0.43, 1.57)	0.55	ns
Gram neg—no. (%)	21 (26.6%)	28 (21.5%)	1.32	(0.69, 2.53)	0.41	1.38	(0.70, 2.75)	0.36	ns
CNS—no. (%)	19 (24.1%)	40 (30.8%)	0.71	(0.38, 1.35)	0.30	0.81	(0.41, 1.60)	0.54	ns
CRP > 45 mg/l—no. (%)	47 (59.5%)	72 (55.4%)	1.18	(0.67, 2.08)	0.56	1.29	(0.70, 2.38)	0.41	ns
Leukocytes < 4 or > 20 × 10^9/L—no. (%)	51 (64.6%)	66 (50.8%)	1.77	(0.99, 3.14)	0.05	1.65	(0.91, 3.02)	0.10	ns
Mechanical ventilation during LOS—no. (%)	58 (73.4%)	66 (46.2%)	3.22	(1.76, 5.91)	< 0.001	2.62	(1.38, 4.96)	0.003	ns
Duration of mechanical ventilation during LOS, days —median (IQR)	5.0 (2.3, 8.8)	2.5 (1.7, 6.2)	1.10	(1.01, 1.19)	0.023	1.09	1.00, 1.18	0.045	ns
Cardiotonics during LOS—no. (%)	22 (27.8%)	23 (17.7%)	1.80	(0.92, 3.50)	0.09	1.36	(0.67, 2.77)	0.40	ns

*a* adjusted for early-onset sepsis (EOS), gestational age (GA), and small for gestational age (SGA). *LOS* late-onset sepsis, *CRP* C-reactive protein, *OR* odds ratio, *aOR* adjusted odds ratio, *CI* confidence interval

Both sensitivity analyses show that that the need for and duration of mechanical ventilation were independently associated with an increased risk for developing BPD as well (Supplemental Table 2). In the culture-proven LOS analysis, this association was also found for abnormal leukocytes count.

## Discussion

To our knowledge, this is the first cohort study investigating which clinical and biochemical characteristics of LOS modulate the development of BPD. Our results show that the need for and the duration of mechanical ventilation during LOS are independent risk factors for the development of BPD. Other clinical and biochemical factors of LOS did not influence the risk of BPD.

Consistent with previous studies, our study shows that infants with LOS have an increased risk of developing BPD compared to those without LOS. Zooming in on the infants with LOS, the only clinical parameter independently associated with the development of BPD was the need and duration of mechanical ventilation. Mechanical ventilation itself is an established risk factor for developing BPD [24, 25]. The same is true for systemic inflammation, which is often present during LOS. Our results suggest that the combination of these two risk factors augments the risk of BPD. Indeed, animal data and studies in neonatal and adult critical care suggest that the combination of systemic inflammation and invasive mechanical ventilation causes more ventilated-induced lung injury than either insult alone [26–28].

Most of the previous studies describing the association between LOS and the development of BPD only included infants with a positive blood culture and/or cerebrospinal fluid culture. However, obtaining an adequate sample for blood culture is challenging in (premature) neonates which results in a large proportion of false-negative blood cultures in clinical infectious infants [14–16]. Therefore, analyzing only culture-proven LOS episodes might introduce a selection bias. For this reason, we included infants with either culture-proven or clinical suspected episodes of sepsis. The fact that we still observed a higher risk to develop BPD makes selection bias in previous studies including only culture-proven sepsis less likely. Reassuringly, the results of the sensitivity analysis including only infants with culture-proven LOS yielded comparable results as previous reports in a similar population.

Previous studies have shown that LOS caused by gram-negative microorganisms is associated with a higher risk of death or BPD compared to those caused by gram positive microorganisms [9]. In this study, we found no modulating effect of microorganism on the risk to develop BPD in the group of infants with a culture-proven LOS. This discrepancy may be due to the relative small number of infants with a positive blood culture in our cohort compared to previous studies.

In contrast to our primary analysis, the sensitivity analysis in culture-proven LOS also showed an association between having leukopenia or leukocytosis and the risk of BPD. The association between leukocytosis and development of BPD has been previously reported in EOS, but not LOS [29]. The fact that we did not observe an association between leukocyte counts and BPD in the primary analysis may have been caused by inclusion of culture negative LOS episodes in this analysis. Some of these infants may have been classified as LOS based on clinical signs, while this was actually not the case (false positive episodes).

The sensitivity analysis including only those infants with one LOS episode showed the same association as found in the primary analysis. In previous studies, an additional risk to develop severe BPD after having repeated LOS episodes has been described [30]. However, this association is described in a specific group of infants receiving mechanical ventilation for 2 weeks. The effect of repeated LOS episodes on the development of BPD can be partly attributed to the duration of mechanical ventilation in their cohort.

The postnatal age of having an infectious episode is a critical determinant of the host immune response to sepsis [21]. Exposure to antimicrobials and stress is known to modify the subsequent immune response and may represent changes associated with trained innate immunity or adaptive immunity [21]. Given this change in immunity, the timing of LOS might alter the risk for long-term complications. This is the first study exploring this possible effect of the postnatal age of LOS onset on the development of BPD, reporting no indication that such a modulating effect exists.

This study has several strengths. It is the first study exploring the association between LOS-associated factors and the development of BPD. In addition, we included all infants treated with antibiotics for culture-proven or clinical suspected LOS episodes. This study also has limitations. First, the data was collected retrospectively, which might have affected the accuracy of the data. Second, since recruitment period of this study was 6 years, residual confounding might have impacted our study results although no major treatment policies were implemented in this period. Third, the data was collected from a single hospital, which might limit the generalizability of the study results. Fourth, in the analyses, we have excluded those infants who died before reaching the age of 36-weeks PMA. No accurate prediction model for the development of BPD is available yet [31]. Therefore, it is impossible to predict if the infants would have been diagnosed with BPD if they had survived. Including all these infants into the BPD group or, in the same line of reasoning, into the no BPD group might lead to an over- or underestimation of the results, respectively. For this reason, we analyzed the results in those infants surviving until 36-weeks PMA. Fifth, in our study, the definition of LOS was based on the duration of antibiotics, regardless of the blood culture. The duration of antibiotic treatment was (according to routine care) at the discretion of the treating clinician, which might be a source of misclassification. However, analyzing the whole group of infants with prolonged antibiotic treatment makes it representative with daily clinical care. One might even consider this inclusion criterion as a strength of this study. Furthermore, the results might be hampered by residual confounding given the potential clinical differences between the two populations, although the pulmonary condition prior to the LOS was not significant different. In addition, during the study period, pulmonary ultrasound was not used as diagnostic tool to rule out a ventilator acquired pneumonia [32]. Therefore, in this study, we cannot differentiate between pulmonary deterioration due to mechanical ventilation versus a ventilation acquired pneumonia secondary to this mechanical ventilation episode. Future prospective studies should incorporate this diagnostic tool in case of pulmonary deterioration after intubation for sepsis. Finally, in this study, no data on whether the infant was exclusively fed with mothers own milk was collected. Previous studies have shown that this factor is an important risk factor in the development of BPD [33, 34]. This covariate should be taken into consideration In future studies.

The results of this study might have implications for clinical practice. Anti-inflammatory agents like corticosteroids are preferably not administered to preterm infants during sepsis, because of the possible suppressive effect on the immune system. Given the results of this study showing the association between need for and duration of mechanical ventilation during a LOS episode and the increased BPD risk, future studies need to investigate if administration of corticosteroids to infants remaining on invasive mechanical ventilation and showing no improvement in lung condition for example 48 h after start of antibiotic treatment reduce the development of BPD in this high risk population.

In conclusion, the need for and the duration of mechanical ventilation during LOS are independently associated with an increased risk of developing BPD in preterm infants below 30 weeks of gestation with LOS.

## Supplementary Information

ESM 1 (DOCX 13 kb)

ESM 2 (DOCX 17 kb)

## Abbreviations

BPD: Bronchopulmonary dysplasia
BW: Birthweight
95% CI: 95% confidence interval
CRP: c-reactive protein
EOS: Early-onset sepsis
GA: Gestational age
IVH: Intraventricular hemorrhage
IQR: Interquartile range
LOS: Late-onset sepsis
NEC: Necrotizing enterocolitis
NICU: Neonatal intensive care unit
OR: Odds ratio
PDA: Persistent ductus arteriosus
PMA: Postmenstrual age
SD: Standard deviation
SGA: Small for gestational age
