# Supplementary material for: The association between clinical and biochemical characteristics of late-onset sepsis and bronchopulmonary dysplasia in preterm infants
Source: Eur J Pediatr. 2021 Feb 25;180(7):2147–54. doi: 10.1007/s00431-021-03981-9 (PMC7904512; doi:10.1007/s00431-021-03981-9)
Supplement: Supplementary file 1 — (DOCX 13 kb) [file 431_2021_3981_MOESM1_ESM.docx]

**Table 1 (Supplemental) –** Patient characteristics of infants with and without LOS.

| **Characteristics** | **LOS**  **n=256** | **No LOS**  **N=500** | **P-value** |
| --- | --- | --- | --- |
| Gender, male – no (%) | 136 (53.1%) | 283 (56.6%) | 0.36 |
| Gestational age – median/IQR | 27.0 (25.7, 28.4) | 28.1 (26.6, 29) | <0.001 |
| Birth weight – g mean / SD | 924 (245) | 1049 (273) | <0.001 |
| Small for gestational age – no. (%) | 47 (18.5%) | 73 (14.8%) | 0.19 |
| Outborn – no. (%) | 20 (7.8%) | 56 (11.3%) | 0.14 |
| Singleton – no. (%) | 182 (71.1%) | 357 (71.4%) | 0.96 |
| Antenatal corticosteroids – no. (%) | 235 (91.8%) | 435 (87.0%) | 0.05 |
| Cesarean section – no. (%) | 112 (44.1%) | 227 (45.7%) | 0.70 |
| PPROM – no. (%) | 50 (19.5%) | 117 (23.4%) | 0.23 |
| Apgar score at 5 min median / IQR | 8 (7, 8) | 8 (7, 9) | 0.30 |
| EOS– no. (%) | 69 (27.0%) | 108 (21.6%) | 0.10 |
| Moderate or Severe BPD – no. (%) | 79 (30.9%) | 48 (9.6%) | <0.001 |
| Intubation at delivery room – no. (%) | 26 (10.2%) | 64 (13.0%) | 0.15 |
| Mechanical ventilation during admission – no (%) | 210 (82.0%) | 239 (47.8%) | <0.001 |
| Mechanical ventilation (days) median/IQR | 5.0 (2.0, 11.0) | 1.3 (0.5, 3.1) | <0.001 |
| CPAP (days) median/IQR | 29.9 (14.1, 40.9) | 8.9 (4.4, 22.2) | <0.001 |
| Surfactant – no. (%) | 125 (49.4%) | 228 (46.0%) | 0.37 |
| Caffeine – no. (%) | 249 (98.0%) | 444 (89.5%) | <0.001 |
| Dexamethasone – no. (%) | 35 (13.8%) | 6 (1.2%) | <0.001 |
| Central venous lines – no. (%) | 240 (93.8%) | 388 (77.6%) | <0.001 |

PPROM: preterm premature rupture of membranes, EOS: early onset sepsis, CPAP: continuous positive airway pressure. IQR: interquartile range, no: number, SD: standard deviation.
