# Supplementary material for: The association between clinical and biochemical characteristics of late-onset sepsis and bronchopulmonary dysplasia in preterm infants
Source: Eur J Pediatr. 2021 Feb 25;180(7):2147–54. doi: 10.1007/s00431-021-03981-9 (PMC7904512; doi:10.1007/s00431-021-03981-9)
Supplement: Supplementary file 2 — (DOCX 17 kb) [file 431_2021_3981_MOESM2_ESM.docx]

**Table 2 (Supplemental)** - Sensitivity analysis of infants with positive blood culture and of infants enduring 1 late onset sepsis.

| **Infants with positive blood culture** | **BPD**  **N= 59** | **No BPD**  **N= 100** | **OR** | **95% CI** | **P-value** |  | **aOR^a^** | **95% CI^a^** | **P-value^a^** |
| --- | --- | --- | --- | --- | --- | --- | --- | --- | --- |
| Timing 1^st^ antibiotics |  |  |  |  |  |  |  |  |  |
| < 7 days – no. (%) | 17 (28.8%) | 33 (32.7%) | 0.83 | (0.41, 1.68) | 0.61 |  | 0.94 | (0.44, 2.04) | 0.88 |
| 7–14 days – no. (%) | 26 (44.1%) | 53 (52.5%) | 0.71 | (0.37, 1.36) | 0.31 |  | 0.69 | (0.35, 1.37) | 0.29 |
| ≥ 14 days – no.(%) | 16 (27.1%) | 15 (14.9%) | 2.13 | (0.96, 4.72) | 0.06 |  | 1.87 | (0.81, 4.31) | 0.14 |
| >1 episode of LOS – no. (%) | 24 (40.7%) | 27 (26.7%) | 1.88 | (0.95, 3.71) | 0.07 |  | 1.46 | (0.69, 3.08) | 0.32 |
| Gram stained bacteria |  |  |  |  |  |  |  |  |  |
| Gram pos – no. (%) | 24 (40.7%) | 40 (40.0%) | 1.03 | (0.53, 1.98) | 0.93 |  | 0.82 | (0.41, 1.66) | 0.59 |
| Gram neg – no. (%) | 21 (35.6%) | 28 (28.0%) | 1.42 | (0.71, 2.83) | 0.32 |  | 1.46 | (0.71, 3.00) | 0.31 |
| CNS – no. (%) | 19 (32.2%) | 40 (40.0%) | 0.71 | (0.36, 1.40) | 0.33 |  | 0.81 | (0.39, 1.67) | 0.57 |
| CRP > 45 mg/l – no. (%) | 43 (72.9%) | 62 (61.4%) | 1.69 | (0.84, 3.40) | 0.14 |  | 2.02 | (0.93, 4.36) | 0.07 |
| Leukocytes <4 or >20x10^9/L – no. (%) | 44 (74.6%) | 55 (54.5%) | 2.45 | (1.21, 4.96) | 0.01 |  | 2.15 | (1.04, 4.47) | 0.04 |
| Mechanical ventilation during LOS – no. (%) | 44 (74.6%) | 47 (46.5%) | 3.37 | (1.67, 6.82) | 0.001 |  | 2.84 | (1.34, 6.02) | 0.006 |
| Duration of mechanical ventilation during LOS, days – median (IQR) | 5.8 (2.4, 9.4) | 2.4 (1.8, 6.3) | 1.11 | (1.02, 1.22) | 0.02 |  | 1.11 | (1.02, 1.22) | 0.022 |
| Cardiotonics during LOS – no. (%) | 18 (30.5%) | 17 (16.8%) | 2.17 | (1.01, 4.64) | 0.046 |  | 1.61 | (0.72, 3.63) | 0.25 |
|  |  |  |  |  |  |  |  |  |  |
| **Infants with one LOS episode** | **BPD**  **N= 51** | **No BPD**  **N= 98** | **OR** | **95% CI** | **P-value** |  | **aOR^a^** | **95% CI^a^** | **P-value^a^** |
| Timing 1^st^ antibiotics |  |  |  |  |  |  |  |  |  |
| < 7 days – no. (%) | 14 (27.5%) | 30 (30.6%) | 0.86 | (0.41, 1.82) | 0.69 |  | 0.86 | (0.37, 1.97) | 0.72 |
| 7–14 days – no. (%) | 21 (41.2%) | 46 (46.9%) | 0.79 | (0.40, 1.57) | 0.51 |  | 0.88 | (0.42, 1.84) | 0.74 |
| ≥ 14 days – no.(%) | 16 (31.4%) | 22 (22.4%) | 1.58 | (0.74, 3.37) | 0.24 |  | 1.35 | (0.60, 3.01) | 0.47 |
| Positive blood culture – no.(%) | 35 (68.6%) | 73 (74.5%) | 0.75 | (0.36, 1.58) | 0.45 |  | 0.87 | (0.39, 1.93) | 0.73 |
| Gram stained bacteria |  |  |  |  |  |  |  |  |  |
| Gram pos – no. (%) | 10 (19.6%) | 27 (27.6%) | 0.64 | (0.28, 1.46) | 0.29 |  | 0.53 | (0.22, 1.28) | 0.16 |
| Gram neg – no. (%) | 15 (29.4%) | 20 (20.4%) | 1.63 | (0.75, 3.54) | 0.22 |  | 1.76 | (0.76, 4.08) | 0.19 |
| CNS – no. (%) | 10 (19.6%) | 27 (27.6%) | 0.64 | (0.28, 1.46) | 0.29 |  | 0.87 | (0.36, 2.10) | 0.75 |
| CRP > 45 mg/l – no. (%) | 24 (74.1%) | 42 (42.9%) | 1.19 | (0.60, 2.34) | 0.62 |  | 1.71 | (0.80, 3.67) | 0.17 |
| Leukocytes <4 or >20x10^9/L – no. (%) | 20 (54.9%) | 44 (44.9%) | 1.49 | (0.76, 2.95) | 0.25 |  | 1.83 | (0.87, 3.83) | 0.11 |
| Mechanical ventilation during LOS – no. (%) | 33 (64.7%) | 35 (35.7%) | 3.30 | (1.63, 6.70) | 0.001 |  | 2.72 | (1.27, 5.84) | 0.01 |
| Duration of mechanical ventilation during LOS, days – median (IQR) | 3.4 (2.0, 7.2) | 2.3 (1.6, 3.8) | 1.22 | (1.01, 1.49) | 0.042 |  | 1.23 | (1.01, 1.51) | 0.043 |
| Cardiotonics during LOS – no. (%) | 9 (17.6%) | 7 (7.1%) | 2.79 | (0.97, 7.99) | 0.06 |  | 2.57 | (0.82, 8.01) | 0.10 |

a = adjusted for early onset sepsis (EOS), gestational age (GA) and small for gestational age (SGA). LOS: late onset sepsis, CRP: C-reactive protein, OR: odds ratio, aOR: adjusted odds ratio, CI: confidence interval.
